# Global Influenza Seasonality: Reconciling Patterns across Temperate and Tropical Regions

**DOI:** 10.1289/ehp.1002383

**Published:** 2010-11-19

**Authors:** James Tamerius, Martha I. Nelson, Steven Z. Zhou, Cécile Viboud, Mark A. Miller, Wladimir J. Alonso

**Affiliations:** 1 School of Geography and Development, University of Arizona, Tucson, Arizona, USA; 2 Fogarty International Center, National Institutes of Health, Department of Health and Human Services, Bethesda, Maryland, USA; 3 London School of Hygiene and Tropical Medicine, London, United Kingdom; 4 British Columbia Institute of Technology, Burnaby, British Columbia, Canada

**Keywords:** contact rates, immunity, influenza, seasonality, virus survival

## Abstract

**Background:**

Despite the significant disease burden of the influenza virus in humans, our understanding of the basis for its pronounced seasonality remains incomplete. Past observations that influenza epidemics occur in the winter across temperate climates, combined with insufficient knowledge about the epidemiology of influenza in the tropics, led to the perception that cool and dry conditions were a necessary, and possibly sufficient, driver of influenza epidemics. Recent reports of substantial levels of influenza virus activity and well-defined seasonality in tropical regions, where warm and humid conditions often persist year-round, have rendered previous hypotheses insufficient for explaining global patterns of influenza.

**Objective:**

In this review, we examined the scientific evidence for the seasonal mechanisms that potentially explain the complex seasonal patterns of influenza disease activity observed globally.

**Methods:**

In this review we assessed the strength of a range of hypotheses that attempt to explain observations of influenza seasonality across different latitudes and how they relate to each other. We reviewed studies describing population-scale observations, mathematical models, and ecological, laboratory, and clinical experiments pertaining to influenza seasonality. The literature review includes studies that directly mention the topic of influenza seasonality, as well as other topics we believed to be relevant. We also developed an analytical framework that highlights the complex interactions among environmental stimuli, mediating mechanisms, and the seasonal timing of influenza epidemics and identify critical areas for further research.

**Conclusions:**

The central questions in influenza seasonality remain unresolved. Future research is particularly needed in tropical localities, where our understanding of seasonality remains poor, and will require a combination of experimental and observational studies. Further understanding of the environmental factors that drive influenza circulation also may be useful to predict how dynamics will be affected at regional levels by global climate change.

Few seasonal disease patterns have generated as much interest, or have so confounded researchers, as the distinct winter epidemics of the influenza virus in temperate populations. Influenza is one of the most significant diseases in humans, considered to be associated with approximately 250,000–500,000 deaths globally each year (World Health Organization 2009).

It remains to be shown what mechanisms are responsible for global patterns of influenza seasonality (Cannell et al. 2006; Dowell 2001; Lipsitch and Viboud 2009; Lofgren et al. 2007). Influenza epidemics in temperate populations occur in the northern and southern hemispheres during their respective winters (Finkelman et al. 2007; Hope-Simpson 1981). Consequently, it has long been speculated that cold temperatures (Davey and Reid 1972), low indoor humidity (Hemmes et al. 1962), and minimal solar radiation (Hope-Simpson 1981) are causally linked to influenza epidemics. Yet in the tropics, where humidity and temperatures remain high year-round, and where solar radiation does not vary strongly (relative to temperate regions), mounting evidence suggests that many of these populations also experience marked seasonal influenza epidemics (Alonso et al. 2007; Dapat et al. 2009; de Mello et al. 2009; Moura et al. 2009; Russell et al. 2008). Further, recent evidence points to a link between increased influenza activity and the rainy season in several tropical populations (Chew et al. 1998; Chumkiew et al. 2007; de Mello et al. 2009; Dosseh et al. 2000; Moura et al. 2009; Rao and Banerjee 1993) when humidity is typically greatest, in contrast to low-humidity (indoor) temperate epidemics. Also in opposition to temperate regions, many tropical populations have significant influenza activity year-round, and some tropical populations are characterized by two distinct influenza seasons, such as Singapore (Figure 1) and Hong Kong (Lee et al. 2009; Viboud et al. 2006a; Yang et al. 2008).

A variety of explanations have been proposed to account for the seasonal nature of influenza. Although many of these explanations have been discussed in past literature (e.g., Cannell et al. 2006; Dowell 2001; Eccles 2002; Lipsitch and Viboud 2009; Lofgren et al. 2007; Mourtzoukou and Falagas 2007), here we review and organize the evidence available for each putative mechanisms in order to identify research gaps (summarized in Tables 1–3). We followed a framework where putative seasonal stimuli drive seasonal influenza incidence through three primary mediating mechanisms: seasonal variations in host contact rate, virus survival, and host immunity (Figure 2) (Dowell 2001; Lipsitch and Viboud 2009; Lofgren et al. 2007). Within this framework, we examined the available evidence regarding each seasonal stimulus, assessed its potential to explain influenza seasonality in both temperate and tropical regions, identify key outstanding questions, and provide recommendations for future research.

## Seasonal Variations in Contact Rates

Increased proximity between susceptible and infected hosts is frequently suggested to be an important driver of influenza seasonality (Table 1). Observations of the rapid dissemination of influenza at the Hajj in Saudi Arabia (Ahmed et al. 2006) and on passenger aircraft (Baker et al. 2010; Moser et al. 1979) and the possibility that crowding among soldiers in World War I hastened the development of the 1918 influenza pandemic (Lofgren et al. 2007) suggest that contact rates are important in influenza virus transmission.

A long-held hypothesis suggests that crowding indoors during cold weather causes wintertime temperate epidemics (Lofgren et al. 2007). A similar mechanism potentially explains the coincident timing between tropical influenza epidemics and the rainy season because individuals may move indoors to escape precipitation. Using a human activity database for various locations in the United States, Graham and McCurdy (2004) demonstrated that individuals spend on average 1–2 hr more indoors during cold weather and spend about 0.5 hr more time indoors during rainy weather. Although these differences are minimal relative to the 21–22 hr individuals spend indoors on average (Graham and McCurdy 2004), it is possible that even a relatively small change in contact and transmission rates could be sufficient to cause epidemics (Dushoff et al. 2004). Accordingly, seasonal variability in contact rates related to school schedules, such as children returning to school from holiday, could drive the seasonal nature of influenza (Lipsitch and Viboud 2009; Lofgren et al. 2007). This is consistent with findings in Cauchemez et al. (2008), which showed that holidays reduce transmission among children in France by 20–29%. In addition, geographical variation in the timing of the fall wave of the 2009 pandemic across the United States has been shown to coincide with geographical variation in school schedules (Chao et al. 2010).

However, although school closures likely have a significant effect on transmission, it has yet to be explained why influenza peaks during the winter in temperate locations, and not during the fall or spring when children are also in school. In addition, it is difficult to imagine how school schedules could result in influenza seasonality in both temperate and tropical locations, because tropical epidemics are sometimes characterized by bimodal seasonality and year-round transmission. Furthermore, crowding also occurs year-round at festivals, sporting events, and conferences without consistent outbreaks of infection (Cannell et al. 2006; Dowell 2001). In addition, in locations such as the deserts of the southwestern United States, hot temperatures regularly drive individuals indoors during the summer, yet these locations are still characterized by winter influenza epidemics.

Therefore, and as has been noted previously (Lofgren et al. 2007), no empirical data link increased contacts rates due to weather conditions and increases in influenza transmission. Nevertheless, it is a strong possibility that variability in contact rates interacts with other seasonal stimuli to determine the precise timing of influenza epidemics. Thus, these mechanisms warrant further investigation. A population-based prospective study using time diaries to investigate the contact characteristics of 7,290 individuals over the course of a single day was a significant step forward in this regard (Mossong et al. 2008 ).

The effect of contact rates at larger spatial scales (e.g., state and continental) on the spread of the influenza virus must also be considered. For instance, Viboud et al. (2006b) demonstrated that human mobility patterns in the United States synchronize epidemics among highly connected populations and that the virus tends to spread from populous to less populous locations. It is possible that contact rates at these larger scales may be particularly important to the seasonal patterns of influenza in smaller populations, or for locations that are characterized by minimal environmental variability. In such cases, seasonal variability in the volume of infected individuals entering a population may exceed any environmentally mediated seasonal forcing. Analysis of seasonality in large-scale human mobility patterns—for instance, using global database on air passenger flows (Hufnagel et al. 2004)—may shed light on this possibility. In addition, it would be extremely interesting to screen year-round incoming air passengers at selected destinations (e.g., islands), collect respiratory samples, and test for seasonal variations in the prevalence of influenza importations.

## Seasonal Variations in Virus Survival

Influenza can be transmitted through several distinct mechanisms, including large droplets, aerosols, and direct contact (including contact with contaminated hosts and surfaces) (Brankston et al. 2007; Tellier 2009; Weber and Stilianakis 2008). To survive during transport among hosts, the influenza virus must be able to endure a variety of environmental conditions. Thus, the effects of the ambient environment on virus survival have long been considered an important factor related to the seasonality of influenza (Table 2) (Hemmes et al. 1962).

Several classical experiments performed in past decades directly measured the tolerance of aerosolized influenza virus to humidity and temperature. Of the six studies that have investigated the issue, four found that influenza virus survival increases monotonically with a decrease in relative humidity (RH) (Harper 1961; Hemmes et al. 1962; Hood 1963; Loosli et al. 1943). The two remaining studies observed a bimodal relationship, with virus survival greatest at low RH, minimal at mid-RH, and moderate at high RH (Schaffer et al. 1976; Shechmeister 1950).

Examining the effect of temperature and RH on aerosol transmission among guinea pigs, Lowen et al. (2007) showed that the efficiency of aerosol transmission decreases as temperature increases from 5°C to 20°C and is completely prevented at 30°C (Lowen et al. 2007). Furthermore, transmission among guinea pigs was inversely related to RH, with high transmission when RH was 20–35% and completely absent at 80% (Lowen et al. 2007). In all, the Lowen et al. (2007) experiments provided evidence that virus survival is important to aerosol transmission, at least in some laboratory settings.

Reexamining available data from the virus survival studies and Lowen et al. (2007), Shaman and Kohn (2009) demonstrated that absolute humidity (AH) is a better predictor (vs. RH and temperature) of influenza virus survival and transmission among guinea pigs. Specifically, virus survival and transmission among guinea pigs increased monotonically with a decrease in AH. This distinction is important because AH is a measure of the amount of water vapor in a volume of air, whereas RH is a measure of the amount of water vapor in the air relative to the amount of water vapor in saturated air of the same volume and air temperature. Further, Shaman et al. (2010) showed that the relationship between AH and virus survival is consistent with observations in the United States where anomalously low AH conditions generally precede the onset of influenza epidemics by approximately 2 weeks. However, temperature and AH are strongly correlated, thereby making it difficult to exclude a confounding effect (Shaman et al. 2010). It is unlikely this mechanism can explain influenza seasonality in the tropics because those regions are typically humid year-round, and epidemics tend to occur during the rainy season, when AH is typically at locally maximal levels. However, as previously stated, there is some evidence that the effect of RH (and potentially AH) on virus survival is bimodal (Minhaz Ud-Dean 2010; Schaffer et al. 1976; Shechmeister 1950), possibly explaining dry-temperate and rainy-tropical epidemics.

Fewer studies have investigated the effects of humidity on the survivability of influenza on surfaces. However, McDevitt et al. (2010) demonstrated that influenza survival on steel surfaces is also inversely related to AH.

The recent findings regarding influenza survival and AH have—at least temporarily—supplanted seasonality explanations regarding the effects of other factors on influenza survival. However, laboratory experiments have shown that the influenza virus becomes more susceptible to inactivation as envelope lipids become increasingly disordered above 21°C (Polozov et al. 2008), suggesting an independent relationship between temperature and virus survival.

Further, several laboratory studies have demonstrated the sensitivity of influenza viruses to ultraviolet radiation (Jensen 1964; Powell and Setlow 1956; Tamm and Fluke 1950). Accordingly, it is hypothesized that viruses expelled into the environment incur lower inactivation rates during seasons with reduced sun activity, consistent with temperate epidemics occurring during the winter and tropical epidemics during the rainy seasons (Sagripanti and Lytle 2007). Hypotheses based on virus survival have to account for the fact that indoor environments, where a great amount of interaction among hosts occurs in modern society, are significantly insulated from external conditions. Because environments often vary considerably between the indoors and the outdoors, the primary location of influenza transmission needs to be determined so that the conditions the virus is exposed to during transmission can be better specified.

## Seasonal Variations in Immunity

Here we broaden the technical definition of immunity to include all physiological functions that enable a host to avoid or mitigate infection after exposure to influenza viruses. The hypothesis that seasonal variation in immunity explains influenza seasonality is supported by observations that humans are less likely to experience influenza-like symptoms after manual inoculation with influenza viruses during interepidemic periods relative to common epidemic months (Shadrin et al. 1977).

Temperature and humidity can affect host immunity through a number of processes (Table 3). For example, the inhalation of cold air causes vasoconstriction in the nose and respiratory tract, resulting in reduced blood flow (Le Merre et al. 1996), diminishing the supply of leukocytes and phagocytic activity in these areas (Eccles 2002; Mourtzoukou and Falagas 2007). Dry conditions can result in moisture losses in the nasal mucosa and reduce mucociliary clearance (Salah et al. 1988). Finally, reactions of host physiology to temperature may alter viral shedding. For instance, Lowen et al. (2007) indicated that peak viral shedding lasted 40 hr longer for guinea pigs housed at 5°C relative to those exposed to 20°C.

Ultimately, although there is sufficient evidence to indicate that human immune function is negatively affected by temperature and humidity, the magnitude of these factors on influenza-specific susceptibility will remain unclear until more laboratory and clinical experiments are performed (Table 3). An example of progress in this regard is a study by Lowen et al. (2007), which indicated that the antiviral and proinflammatory responses of guinea pigs housed at 5°C and 20°C were similar, suggesting that the innate immune response is not impaired at low temperatures. Although this experiment could have been more complete by testing the first line of host defenses, such as mucociliary function, it is a useful example of how laboratory experiments can shed light on seasonal variations in immunity and their impact on virus replication and transmission.

The duration of human exposure to solar radiation, or “photoperiod,” may also modulate immunity at seasonal time scales related to host vitamin D status (Cannell et al. 2006). Human vitamin D levels are generally dependent upon exposure to ultraviolet B radiation, and in turn, deficiencies of this vitamin are common in temperate populations during the winter when solar radiation is lowest (Brustad et al. 2007; Holick 2006; Holick et al. 2007; Webb et al. 1988). Vitamin D has been demonstrated to stimulate innate immunity (Abu-Amer and Bar-Shavit 1993; Gombart et al. 2005; Helming et al. 2005; Liu et al. 2006; Wang et al. 2004). Two studies have shown that individuals with lower vitamin D levels are significantly more likely to report respiratory infections (Aloia and Li-Ng 2007; Ginde et al. 2009). Furthermore, a recent randomized, controlled study to test the effect of vitamin D supplementation on influenza A and B incidence in school children indicated that the controls were significantly more likely to become infected with influenza A than the experimental group (Urashima et al. 2010). However, another randomized, controlled study found no significant association between vitamin D supplementation and respiratory infections (Li-Ng et al. 2009), and Urashima et al. (2010) reported no significant effect of vitamin D supplementation status and influenza B infection in school children. Less is known about vitamin D levels in tropical latitudes where solar radiation varies less substantially over a year, but seasonality in vitamin D levels has been reported in subtropical Hong Kong (MacDonald and Swaminathan 1988). The lowest levels of solar radiation in the tropics typically coincide with the local rainy seasons due to cloudiness. This appears consistent with the relationship between tropical epidemics and the rainy season. Clothing, skin pigmentation, age, behavioral habits, and other factors also affect vitamin D status (Nowson and Margerison 2002), providing opportunities to investigate how individual levels of vitamin D in the same localities and season are correlated with influenza occurrence.

Other nutrients are also required for proper immune function and can modify the pathology of infection. This opens the possibility that seasonal variations in nutrient availability and/or requirements on diet might also have a role on influenza seasonality. Experimental studies in mice suggest that the supplementation of vitamin C (Li et al. 2006) and vitamin E (Han et al. 2000; Mileva et al. 2002) can diminish the severity of influenza infection. Selenium deficiency has also a negative effect on host immune response and the severity of infection in human airway epithelial cells (Jaspers et al. 2007). Hamer et al. (2009) indicated a statistically significant association between general micronutrient deficiency and respiratory disease in elderly individuals in Quito, Ecuador. In all, this hypothesis may be most viable in low-income communities because seasonal effects on local food availability and diet are more difficult to compensate for with other nutritional sources. Variations in dietary intake of poorer individuals in developed communities may occur because of seasonal financial pressures, such as those caused by the cost of heating during temperate winters (Bhattacharya et al. 2003). Ultimately, it is unlikely that the seasonal variability of diet is sufficiently widespread and severe to account for global influenza patterns; however, it may be important in some populations.

Several understudied hypotheses regarding seasonal variations in human immunity also warrant mentioning. For instance, there is an abundance of information on the effects of photoperiod and dark/light cycles on physiology, affecting the immune systems of mammals, including humans, possibly mediated through secretions of melatonin (Dowell 2001). Seasonal changes in airborne particulate matter and pollutants may also have a detrimental effect on immune function (Zhou 2009). Increases in the energy required for thermoregulation may limit the energy available for immune function (Lochmiller and Deerenberg 2000). This may explain influenza epidemics during temperate winter conditions or during damp conditions related to the rainy season in the tropics. The effects of abrupt changes in temperature may also be detrimental to immune function (Bull and Morton 1978).

Another immune mechanism that needs consideration is the interaction among influenza and other pathogens. Host cells produce and release interferons in response to many viral infections, resulting in a decrease in susceptibility to subsequent viral challenges. This heightened state of immune activity during and after a viral epidemic increases herd immunity, making it difficult for additional viruses to become established in a population (Ånestad 1987). In temperate locations, the influenza season overlaps with periods of infection by other viral pathogens, such as rhinoviruses and respiratory syncytial viruses. Observational studies suggest that the timing of non-influenza epidemics can modify the timing of influenza epidemics. For instance, during the fall of 2009, a rapid decline of laboratory-confirmed H1N1 influenza cases in Sweden (Linde et al. 2010) and an unexpectedly slow start to the H1N1 epidemic in France both coincided with rhinovirus outbreaks (Casalegno et al. 2010). It is less clear how influenza covaries with other viruses in tropical regions. Yet, although pathogen interactions may explain subseasonal variations of influenza incidence, it likely does not explain why influenza viruses (and other co-circulating viruses) preferentially spread during temperate winters and tropical rainy seasons.

## Discussion

A major outstanding question regarding influenza seasonality is whether a single seasonal stimulus (or set of stimuli) accounts for universal patterns in influenza virus activity, or whether the key mechanisms underlying the seasonality of influenza in temperate regions differ from those in the tropics. Currently, the most accepted hypotheses explaining influenza seasonality, such as AH and virus survival, attempt to explain influenza seasonality only in temperate regions. Less attention has been given to influenza seasonality in the tropics, likely because of the lack of information about seasonal signals in the past. To advance our understanding, it is important either that hypotheses explaining temperate influenza seasonality are amended so that they include the tropics, or that hypotheses are generated specifically for the tropics that will work in harmony with temperate explanations. To this end, identifying where temperate (cold and dry) and tropical (rainy) relationships with influenza epidemics break down geographically and how this relates to relevant environmental variables (e.g., temperature, humidity) is a short-term and practical goal.

Although numerous mechanisms have been proposed to account for global patterns of influenza seasonality, distinguishing the causal relationships from colinear and confounding associations has proved overwhelming. To overcome this challenge, rigorous observational and experimental studies will be necessary. Laboratory studies are crucial, because confounding factors can be isolated and controlled, and their effects on host immunity and/or virus survival can be specifically addressed and properly measured. Ecological studies should be used to assess the consistency between the results of experimental and observational studies and influenza seasonality observed globally. When possible, it is important that studies use laboratory-confirmed influenza data so that influenza-specific factors are identified, versus those related to other infections with similar symptomology.

Finally, no understanding of influenza seasonality is complete without consideration of the intrinsic aspects of the disease and the ability of the virus to continually reinfect humans by escaping recognition by host antibodies via frequent mutation, a process of selectively driven evolution termed “antigenic drift” (Webster et al. 1982). According to Dushoff et al. (2004), antigenic drift and the subsequent waning immunity of a population provide a seasonal forcing that is nearly sufficient to stimulate seasonal influenza epidemics each year (“dynamic resonance”), requiring only relatively minor changes in extrinsic seasonal forces to “lock” epidemics into phase. However, the mathematical model used to support this hypothesis relies upon unrealistically large rates of reproduction for the influenza virus (Andreasen et al. 2008; Chowell et al. 2007; Viboud et al. 2006b) and oversimplifies important evolutionary questions related to antigenic drift and partial cross-immunity among strains. Moving forward, more studies, conducted in parallel with a robust description of antigenic changes in circulating viruses, are needed that explore changes in population immunity and virus transmissibility over time, both between seasons and during the course of an epidemic. These studies would allow a better understanding of the intrinsic dynamics of influenza viruses and would help determine the contribution of dynamic resonance to influenza seasonality patterns.

In addition to increasing our capacity to design effective public health prevention and control interventions (Lipsitch and Viboud 2009), understanding the seasonal processes associated with influenza seasonality will potentially inform us about the processes related to the seasonality of other infectious diseases. Further, the understanding of the environmental factors that cause influenza seasonality should also be useful to forecast novel dynamics in regional contexts due to changing environmental and climatological scenarios. Thus, this is an important scientific and public health endeavor. Ultimately, the more systematically we proceed in our investigation of influenza seasonality, the more likely we are to unravel this age-old epidemiologic mystery.

## Figures and Tables

**Figure 1:**
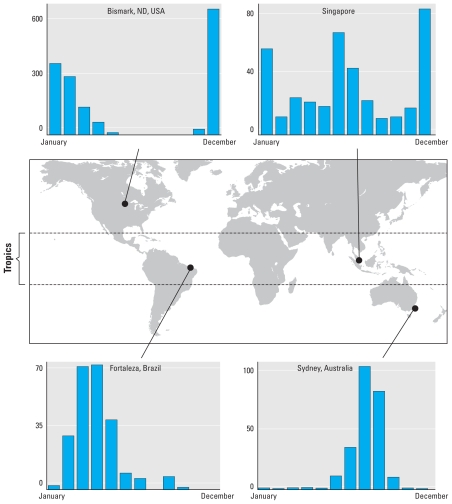
Seasonal patterns of influenza in four sites across several latitudes worldwide. Temperate epidemics occur predominantly during the winter months, when the environment is cool and dry and solar radiation is low. Seasonal influenza activity in the tropics appears to be greatest during the rainy season. The bar charts indicate the average number of detected influenza isolates (*y*-axis) over several years for Singapore (Chew et al. 1998), Fortaleza, Brazil (Moura et al. 2009), Bismarck, North Dakota, USA (Irmen and Kelleher 2000), and Sydney, Australia (Keflemariam et al. 2004).

**Figure 2:**
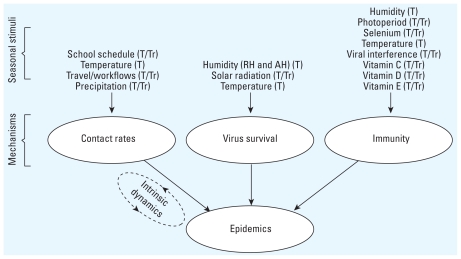
Putative relationship and causal connections among key seasonal stimuli, mediating mechanisms, and influenza epidemics. The notation adjacent to each seasonal stimulus indicates whether it potentially explains influenza seasonality in the tropics (Tr), temperate regions (T), or both (T/Tr). The diagram also includes a component depicting the effects of intrinsic dynamics.

**Table 1:** Contact rates and influenza seasonality.

Predictor	Outcome^a^	Key studies^b^	Conclusions
School closures	Contact rates	O: Hens et al. 2009 O: Miller et al. 2010	In general, contact among children declines significantly during weekends and holidays and varies significantly by age group.
	Influenza transmission	O: Kar-Purkayastha et al. 2009 A: Kawaguchi et al. 2009	Influenza transmission frequently occurs among children in schools.
	Influenza rates	Ec: Cauchemez et al. 2008 Ec: Cowling et al. 2008 MM: Glass et al. 2007 MM: Lee et al. 2010 Ec: Chao et al. 2010 Ec: Heymann et al. 2009	School closures can have a significant impact on influenza transmission among children, and the return of children to school may catalyze epidemics.
Temperature	Contact rates	O: Graham and McCurdy 2004	Individuals spend 2 more hours per day on average indoors during cold days, potentially increasing contact rates.
	Influenza rates	Ec: Dosseh et al. 2000 Ec: Urashima et al. 2003	In many temperate regions, influenza is associated with cool temperatures. However, this is not always the case in tropical and subtropical locations.
Travel/work flows	Influenza transmission	O: Moser et al. 1979 O: Baker et al. 2010	There is evidence that influenza can be transmitted on passenger airlines.
	Influenza rates	Ec: Brownstein et al. 2006 Ec: Viboud et al. 2006b	Travel may synchronize epidemics among highly connected populations.
Precipitation	Contact rates	O: Mikolajczyk et al. 2008	There is a significant decline in the number of contacts among school children during rainy days.
	Influenza rates	Ec: Moura et al. 2009 Ec: Urashima et al. 2003	Tropical influenza epidemics tend to occur during the rainy season. However, there is no clear association between temperate locations and epidemics.

a Studies describing “influenza transmission” document transmission of influenza among humans or other hosts. Studies describing “influenza rates” describe rates of influenza or proxy indicators (e.g., upper respiratory illness, influenza-like illness, pneumonia, and influenza morbidity) in a population.

b Letter codes indicate the type of study: A, anecdotal; E, experimental studies where the researcher manipulates variables in an attempt to determine their effects on the outcome of interest; Ec, ecological studies where the unit of analysis is a population rather than an individual; M, meta-analysis studies where the researcher combines information from several studies to draw conclusions; MM, mathematical modeling studies where the researcher creates a mathematical algorithm to describe the system of interest, and manipulates parameters to observe their effects; O, observational studies where the researcher observes associations between outcomes of individuals and variables.

**Table 2:** Virus survival and influenza seasonality.

Predictor	Outcome	Key studies	Conclusions
Humidity	Virus survival	E: Harper 1961 E: Hemmes et al. 1962 E: Hood 1963 E: Loosli et al. 1943 E: McDevitt et al. 2010 E: Schaffer et al. 1976 M: Shaman and Kohn 2009 E: Shechmeister 1950	Influenza virus survival increases as AH (and RH) humidity decreases both in aerosol and on surfaces. AH has been shown to be the best predictor of virus survival.
	Influenza transmission	E: Lowen et al. 2007 E: Lowen et al. 2008 M: Shaman and Kohn 2009	Influenza transmission by aerosol among guinea pigs is most efficient in low AH (and RH) conditions; transmission of influenza via short-range contact was not affected by humidity (AH or RH).
	Influenza rates	Ec: Shaman et al. 2010 Ec: Tang et al. 2010	There is evidence that decreases in AH may catalyze seasonal influenza epidemics in temperate locations, but this does not hold for tropical locations.
Solar	Virus survival	E: Jensen 1964 E: Powell and Setlow 1956 E: Tamm and Fluke 1950	Influenza is inactivated by ultraviolet radiation.
Temperature	Virus survival	E: Polozov et al. 2008	Virus survival decreases as temperature increases.
	Influenza transmission	E: Lowen et al. 2007 E: Lowen et al. 2008	In guinea pigs, aerosol transmission of influenza is most efficient at low temperatures; transmission via short-range contact is not affected by temperature.
	Influenza rates	See Table 1	

See notes in Table 1 for discussion of outcomes and study abbreviations.

**Table 3:** Immunity and influenza seasonality.

Predictor	Outcome	Key studies	Conclusions
Humidity	Immune function	E: Salah et al. 1988	There is evidence that inhalation of dry air inhibits mucociliary clearance.
	Influenza rates	See Table 1	
Photoperiod	Immune function	E: Blom et al. 1994 E: Demas et al. 1998 E: Yellon et al. 1999	Mammal hosts may use photoperiod to regulate immune function and anticipate seasonal stress.
Selenium	Immune function	E: Beck et al. 2001 E: Jaspers et al. 2007	Studies have shown that the severity of influenza infection is greater in selenium-deficient mice than in selenium-adequate mice.
Temperature	Immune function	E: Baetjer A 1967	There is some evidence that inhalation of cold air inhibits mucociliary clearance.
	Influenza rates	See Table 1	
Viral interference	Influenza rates	Ec: Ånestad 1987 Ec: Casalegno et al. 2010 Ec: Linde et al. 2010 O: Nisii et al. 2010	There is evidence that cocirculating viruses can delay the onset of influenza epidemics.
Vitamin C	Immune function	E: Li et al. 2006	There is evidence that vitamin C supplementation in mice mitigates influenza infection.
Vitamin D	Immune function	E: Abu-Amer and Bar-Shavit 1993 E: Gombart et al. 2005 E: Helming et al. 2005 E: Liu et al. 2006 E: von Essen et al. 2010 E: Wang et al. 2004	There is strong evidence that vitamin D regulates antimicrobial innate immune responses.
	Influenza rates	O: Aloia and Li-Ng 2007 O: Ginde et al. 2009 E: Li-Ng et al. 2009 E: Urashima et al. 2010	There is evidence that vitamin D levels and vitamin D supplementation may have protective effects against influenza and other respiratory infections in humans.
Vitamin E	Immune function	E: Han et al. 2000 E:Mileva et al. 2002	Vitamin E supplementation may diminish severity of influenza infection in mice.

See notes in Table 1 for discussion of outcomes and study abbreviations.
